# Explainable Artificial Intelligence for Bias Detection in COVID CT-Scan Classifiers

**DOI:** 10.3390/s21165657

**Published:** 2021-08-23

**Authors:** Iam Palatnik de Sousa, Marley M. B. R. Vellasco, Eduardo Costa da Silva

**Affiliations:** Department of Electrical Engineering, Pontifical Catholic University of Rio de Janeiro, Rio de Janeiro 22453-900, Brazil; marley@ele.puc-rio.br (M.M.B.R.V.); edusilva@ele.puc-rio.br (E.C.d.S.)

**Keywords:** computer vision, Computerized Tomography, Covid 19, Explainable AI, image classification, medical imaging

## Abstract

Problem: An application of Explainable Artificial Intelligence Methods for COVID CT-Scan classifiers is presented. Motivation: It is possible that classifiers are using spurious artifacts in dataset images to achieve high performances, and such explainable techniques can help identify this issue. Aim: For this purpose, several approaches were used in tandem, in order to create a complete overview of the classificatios. Methodology: The techniques used included GradCAM, LIME, RISE, Squaregrid, and direct Gradient approaches (Vanilla, Smooth, Integrated). Main results: Among the deep neural networks architectures evaluated for this image classification task, VGG16 was shown to be most affected by biases towards spurious artifacts, while DenseNet was notably more robust against them. Further impacts: Results further show that small differences in validation accuracies can cause drastic changes in explanation heatmaps for DenseNet architectures, indicating that small changes in validation accuracy may have large impacts on the biases learned by the networks. Notably, it is important to notice that the strong performance metrics achieved by all these networks (Accuracy, F1 score, AUC all in the 80 to 90% range) could give users the erroneous impression that there is no bias. However, the analysis of the explanation heatmaps highlights the bias.

## 1. Introduction

The COVID-19 outbreak has become a central topic of research and public discussion throughout 2020 and 2021, after millions of cases and deaths around the world [1]. A key problem regarding this disease is the fast spread and the lack of reliable testing (such as using reverse transcription, or RT-PCR, tests) in enough numbers, in many locations [1]. Additionally, the magnitude of this pandemic results in overworked medical staff in places with less testing capabilities.

This situation greatly motivated the use of Computer Assisted Diagnostic (CAD) tools, to help medical professionals in the triage and sorting of cases. Two main approaches along this line include classification of Chest X-Rays (CXR) and Computerized Tomography (CT) scans, in order to identify abnormal structures that can be correlated to the presence of SARS-CoV-2.

Throughout the pandemic, a growing number of works have been published, describing CXR and CT-scan models for the tasks of multiclass classification of CXR and CT-scan images [2,3,4]. Typically, the problem consists in images of two classes (COVID vs. healthy), three classes (COVID vs. healthy vs. other chest diseases), or more classes discerning into more specific chest diseases. A number of such publications report promising classification metrics [5,6,7]. Generally, the models are based on various types of Convolutional Neural Networks (CNNs), which are currently the best type of image classifiers for medical imaging data.

Although it seems to be generally agreed upon in literature that CT-Scans allow for better discernment and diagnosis for this particular disease [1,8], the majority of works focuses on CXR, perhaps due to the more immediate availability of CXR in general.

However, composing an adequate image dataset was a major issue for these studies towards the development of COVID classifiers using CNNs, due to the unavailability and scarcity of imaging data in large quantities. In this context, initial efforts involved creating datasets through automatic crawling of COVID related papers and since then the resulting datasets have been combined and augmented [3]. Furthermore, aiming at creating more complete datasets, that have images of other chest diseases besides COVID, pre-covid datasets are used and mixed with data from different publications, captured with different devices. Consequently, the resulting datasets usually have images with different aspect ratios and color schemes, among other characteristics.

Such aspects raise concerns over the potential biases these datasets may be introducing onto classifiers. As of yet, few publications seem to address this problem beyond passing comments. As a counterpoint, Catala et al. [8] study the specific case of a VGG16 classifier [9] trained on a CXR datasets. The author used GradCAM [10] heatmaps to visualize biases in classifications.

Indeed, Explainable Artificial Intelligence (XAI) techniques such as GradCAM are arguably ideal tools for probing black-box classifiers, and attempting to extract information regarding the classification process. Moreover, they can be fundamental assets in identifying biases.

Particularly, this study aims at addressing a number of gaps in literature, given the previously discussed scenario. The contributions include:

1. Applying several XAI techniques (GradCAM, LIME, RISE, Squaregrid, Vanilla, Integrated, Smooth Gradients) to compare their resulting heatmaps, and evaluate what biases they expose for the studied classifiers. To our knowledge, this is the first publication that addresses this comparison in a real use case for bias detection in a medical image classifier.
2. Analyzing the influence of the architecture choice in classification bias. Namely, this study compares classifiers of the DenseNet, VGG and EfficientNet families. It also compares how these models behave at different stages of validation, as well as the effect of ensembling.
3. Analyzing the less explored CT-Scan classifiers and datasets, rather than the already more commonly analyzed CXR ones.

As such, the primary focus of this project is XAI, while analyzing the behaviors of different architecture types in regards to influence from different aspects of the images in the dataset.

In this sense, this work also contributes to the increasingly more discussed necessity of transparency and interpretability of AI systems in medicine overall [11,12].

## 2. Materials and Methods

This section describes the dataset, the CNN models and the employed XAI techniques.

### 2.1. Dataset

The dataset used for this paper is the COVID-CT dataset provided by Yang et al. [1]. It consists of images extracted from various COVID-19 related papers, containing a total of 746 images divided into training, validation and test sets of 425, 118 and 203 images, respectively. The images are divided into two classes, NonCOVID (class 0) and COVID (class 1), with the following distribution:

- Training set: 234 class 0 and 191 class 1,
- Validation set: 58 class 0 and 60 class 1
- Test set: 105 class 0 and 98 class 1.

Therefore, it is noticed that there are not significant imbalances, at least in the amount of examples for each class. Further details about the crawling process used for generating this dataset are described in [1].

**Figure 1:**
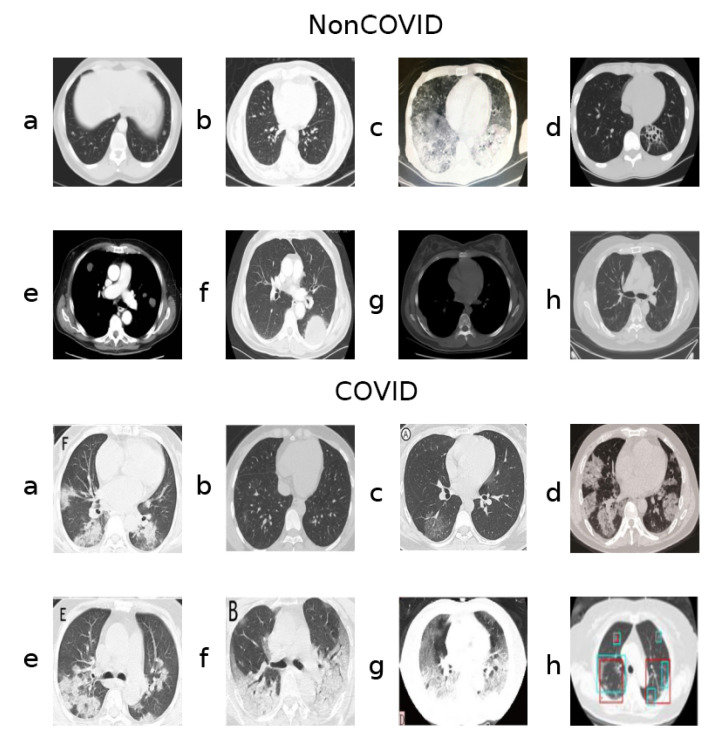
Samples from the two classes (NonCOVID and COVID).

A key aspect of this dataset, however, is that, although classes are roughly balanced considering the number of samples, they do seem to present unequally frequent artifacts and other such image aberrations. Figure 1 shows samples from both classes.

Notably, the samples shown from the COVID class contain letters from annotations (Figure 1, COVID class, panels **a**, **c**, **e**, **f**, **g**) and even colored markings (Figure 1, COVID class, panels **g** and **h**). Sometimes the images are also not presented in a gray-scale map, containing hues of different colors (Figure 1, COVID class, panel **d**), although the majority of the dataset is gray-scale.

Many images in both classes also contain a round frame surrounding the body within the picture, as well as a visible support structure over which the body is laying during the CT-scan. Additionally, there are varying contrast/lightning conditions and aspect ratios, possibly because these CT-scans were captured form different devices and the images were taken by different operators.

### 2.2. Models

#### 2.2.1. DenseNet

Typically, in a CNN the feature maps of a convolution are only used as input in the next convolution. The DenseNet family of architectures expands upon this idea by presenting the output of a convolution to all the subsequent convolutions. This encourages feature reuse and has been shown to lead to strong performances for image classification [13].

These connections across all convolutions also decrease vanishing gradient problems despite the large depth of the architectures, and the feature reuse allows for a greatly decreased number of parameters compared to other networks of similar depths.

Yang et al. [1] have employed a DenseNet169 [13] architecture for the classification task discussed in Section 2.1. Afterwards, He et al. made the weights for this model, pre-trained on Imagenet [14], available on a public repository [15,16]. The following performance metrics are reported in the repository [16]:

- F1 score: 0.854
- Accuracy: 0.847
- AUC: 0.919

The files on this repository are intended for the PyTorch deep learning framework. However, the XAI methods discussed further ahead in this paper were already implemented for Tensorflow. Then, an important step was to try and recreate these results by employing a DenseNet169 on this latter platform.

#### 2.2.2. VGG

As a counterpoint to DenseNet models, it would be interesting to study other representative state-of-the-art architectures and compare the XAI heatmaps generated by their classifications. The VGG architecture [17], namely, VGG16, was chosen for this purpose, because it has been previously probed for CXR classification biases [8] and it is a commonly used high performance classifier for chest radiography type images in general [18,19,20].

The VGG models are well established image classifiers composed by convolutional and pooling blocks followed by a densely connected classification block. The convolutional blocks include filters in increasing numbers, with the convolutions closer to the output typically extracting the features with higher abstractions of the data.

#### 2.2.3. EfficientNet

To further enrich this comparison, the latest state-of-the-art model for image classification was included in this study. The EfficientNet family of classifiers [21] optimize the architecture width, depth and resolution in order to maximize performance for a given computational resource bottleneck. They have been shown to outperform previous architectures on datasets such as CIFAR-100 and other commonly used transfer-learning datasets, while using less parameters than other high performance models, by a full order of magnitude [21]. The basic structure of EfficientNets is similar to other CNNs, relying on successive convolutions, but the number of filters and width of the layers is extracted from an automated neural architecture search [21].

#### 2.2.4. Ensemble

Asides from using the aforementioned architectures, it was also interesting to check whether creating ensembles from them could increase the classification metrics. It was also interesting to test what ensembling would change, from an XAI perspective.

These models were combined in all possible ensembles that could be formed (16,383 in total), and their respective performance metrics were computed on the validation set. This processes was used to identify the ensemble that leads to the best performance metrics. Besides, it is worth mentioning that if more than one ensemble reached the same performance, the adopted tie-break criterion was to select the ensemble with fewer models.

The ensembled prediction is the simple average over the predictions of the models in the ensemble. All models contribute with equal weight to this average. This is the case for all ensemble results described in this work.

#### 2.2.5. Training

After preliminary tests, the size of the analyzed dataset seemed to be insufficient for convergence, when training the models from scratch. However, better performance was reached by using transfer-learning from Imagenet, which is a common procedure for this type of classification.

The models were then trained for 200 epochs, and saved each time a given weight update provided a validation accuracy (*acc*) above 80%. The idea was to find which of these intermediary snapshots of the training process could provide the best ensemble classifier by combining the DenseNet, VGG and EfficientNet models. In total, 14 models were obtained with validation accuracies above the threshold set (5 DenseNet, 5 VGG and 4 EfficientNet).

All architectures were trained using a transfer learning approach (Imagenet weights) and real time data augmentation (flip, rotation, translation and zoom). The models were initially trained for 30 epochs freezing all weights, except for the classification layers, using the base architectures (VGG, EfficientNet, DenseNet) as feature extractors. After this initial step, the models were entirely unfrozen (all layers are made trainable) and training went on for 1000 epochs with a checkpointing approach, that saves any model weights giving validation accuracies above 80%. The hyper-parameters (batch size, number of training epochs, data augmentation parameters, etc) were empirically set to achieve similar performance to He et al. [15,16] (see Section 2.2.1), without necessarily doing extensive fine tuning to try and surpass that performance. The main goal was to create similarly strong classifiers that can be studied with XAI methods, rather than necessarily creating a new state of the art classifier. Indeed, as it can be seen in Section 3, this goal was achieved.

### 2.3. Explainable AI

**Figure 2:**
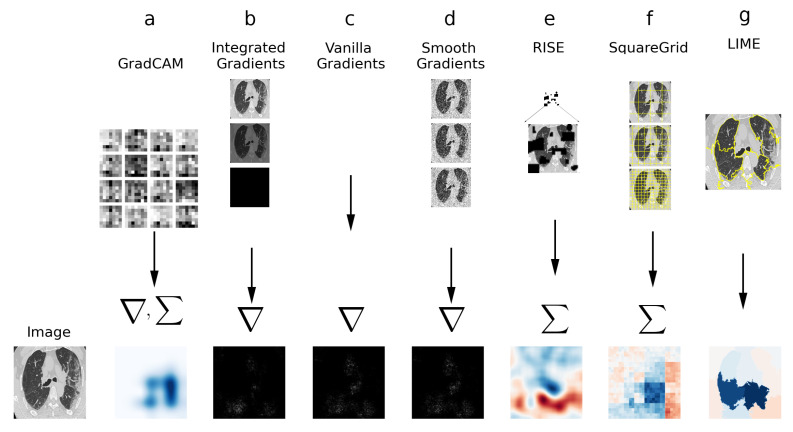
Visual diagram of XAI techniques used in this study. (**a**) GradCAM: gradients to the last convolution are computed and weighted sum is calculated. (**b**) Integrated Gradients: Sum/integral based on interpolations of the image. (**c**) Vanilla Gradients: Direct gradient computation. (**d**) Smooth Gradients: gradient computation on noisy versions of the image. (**e**) RISE: weighted sum of perturbation masks. (**f**) Squaregrid: perturbations using square grid divisions. (**g**) LIME: perturbations using superpixels computed via SLIC.

In this section, the XAI techniques employed for this study are described. Figure 2 contains a simplified visual diagram of the techniques, with their defining features.

#### 2.3.1. LIME

LIME was described for the first time in 2017 by Ribeiro et al. [22]. It can be used for black box classifiers as a whole. In this paper, however, it is used specifically for image classifiers.

The general principle of this technique for explaining the classification of a particular image can be summarized in the following steps:

- The image is segmented into superpixels. These are regions of similar colors/textures that hold some type of visual context;
- A distribution of perturbed images is created from the segmented original image, by stochastic perturbation of the original;
- The perturbed images are presented to the classifier model, and their prediction probabilities are computed;
- With the perturbed distribution and prediction probabilities, a surrogate linear model is trained to approximate the CNN locally for the image of interest;
- The weights of this linear model indicate how much each superpixel contributes positively or negatively to the classification;
- These weights are plotted on a heatmap, which is the final output of LIME.

The segmentation algorithm chosen for this study was the Simple Interactive Linear Clustering (SLIC), developed by Achanta et al [23]. SLIC was empirically chosen among the base segmentation algorithms included in the LIME libraries, after initial tests performed to evaluate which segmentation algorithm could better segment the lungs, or parts of the lungs, from the surrounding background.

The SLIC algorithm represents an image in a 5D space, combining the spatial dimensions of the image with the colors in the CIELAB color space [23] and performing k-means clustering. The main control variable is the approximate number of superpixels desired ($n_{slic}$), that is passed to the algorithm in order to influence the clustering. For this study, preliminary testing lead to a heuristic of $n_{slic}=15$, which generally satisfactorily differentiates the background from the lung areas.

#### 2.3.2. Squaregrid

As a means of avoiding segmentation parameter fine tuning and heuristics, a more coarse approach, developed by [24], was also analyzed, where square grids are used instead of superpixels. Although the resulting heatmaps are less defined, they do not require arbitrary parameter choices and can provide a rough localization of zones of interest in the heatmaps. This method is named Squaregrid.

In this study, the grids are divided in powers of 2, with the grids being centered so that any offsets are localized towards the edges of the image. To justify this choice, it is assumed that the regions of interest are typically closer to the center of the image, rather than their edges.

Each grid is passed to LIME as a segmentation in order to generate the intermediate explanatinos, and their contributions are then summed.

Although on average Squaregrid explanations demand a larger computation time than a single individual LIME explanation, they offer a great advantage compared to running hundreds or thousands of LIME explanations, while attempting to fine tune segmentation parameters.

#### 2.3.3. RISE

In 2018, Petsiuk et al. [25] published another approach of explanations by perturbing inputs, called Randomized Input Sampling for Explanation (RISE). Similarly to LIME, RISE is also a model agnostic approach, which can be applied to any type of black box image classifier. However, there are two main differences between these techniques. Instead of perturbing superpixels and using surrogate models to approximate the explanations locally, RISE is based on the use of stochastically generated masks that cover the original image [25]. Each mask M receives a weight that corresponds to the *softmax* classification probability for the image I perturbed by the mask, $I_{perturbed}=I⊙M$, where the symbol ⊙ denotes a term by term multiplication between the *arrays*.

As such, the final *heatmap*, H can be computed as [25]:

(1) $$H_{RISE}\;\underset{MC}{\approx }\;\frac{1}{p_{1}N}\sum_{i}^{N}f\left(I⊙M_{i}\right)M_{i}$$

where the sum is defined over all generated *N* masks, *f* is the black-box model to be explained (that is, the CNN), and $p_{1}$ is the probability that a pixel is preserved by the masks.

Petsiuk et al. mention that generating masks pixel by pixel at the original image’s dimension is computationally unfeasible, with an added risk of creating undesirable adversarial effects. As such, they suggest generating small masks, which are then upsampled bilinearly until the desired dimension [25].

#### 2.3.4. GradCAM

Arguably the most used and cited Saliency XAI technique, Gradient-weighted Class Activation Maps (GradCAM) was first published in 2016 by Selvaraju et al [10].

GradCAM consists in a saliency technique based on activation maps, drawing features from the last convolution before the classification block in CNNs. The basic principle of GradCAM consists in calculating the gradient of a given output neuron and computing this gradient backwards towards the last convolution. The assumption is that this last layer before classification contains the deepest and most detailed abstractions learned by the network, meaning that it would then generate the most interpretable heatmaps [10].

As such, for a given class *c*:

(2) $$\alpha_{c}^{k}=\frac{1}{Z}\sum \nabla_{k}y^{c}$$

where $y^{c}$ represents the specific output of a class *c* in the CNN, *k* represents a feature map in the last convolution of the CNN, Z is the total number of pixels in this feature map and the sum is defined over all the dimensions of this map. Additionally, $\alpha_{c}^{k}$ is a number that represents the influence of the weight of each feature map *k* over the output neuron corresponding to class *c*.

The sum over all pixels in the feature map, divided by the total number of pixels, is also known as Global Average Pooling (GAP). According to Selvaraju et al. [10], the choice of GAP over other average or pooling options was done empirically, since it generates the best results.

Finally, the heatmap $H_{GradCAM}$ may then be obtained by the linear combination of feature maps, where the weighs of this linear combination are $\alpha_{c}^{k}$, as calculated in Equation (3).

(3) $$H_{GradCAM}=\mathrm{ReLU}\left(\sum_{k}\alpha_{c}^{k}A^{k}\right)$$

Selvaraju et al. use a ReLU activation to eliminate negative contributions to the class of interest, mentioning that negative pixels most likely belong to other classes.

Importantly, note that the generated heatmap has the same dimensions of the last convolution feature maps, which typically will be smaller than the original image. Consequently, the map is resized to the original image size through bilinear upsampling [10].

#### 2.3.5. Vanilla Gradients

Another approach to generating heatmaps involves directly computing the gradients from an ouput neuron back to each input pixel. This straightforward method is often referred to as Vanilla Gradients, and was originally published by Simonyan et al. [17] in 2013.

Notably, the heatmaps generated with this technique only show which pixels were relevant in some way to the classification, without specifying if the contribution was positive or negative.

#### 2.3.6. Smooth Gradients

In 2017, a more sophisticated version of Vanilla Gradients was published by Smilkov et al. [26]. The authors mention that typically the Vanilla Gradients heatmaps contain a lot of noise, most likely caused by local fluctuations in the output activation derivatives. In order to smooth out these fluctuations, they propose computing gradients for several versions of the original image, perturbing them with gaussian noise.

Given the lack of a specific quantitative metric to compare Smooth to Vanilla gradients, the authors mention that qualitatively the results appear to have less noise.

#### 2.3.7. Integrated Gradients

In addition, in 2017, Sundararajan et al. proposed another way to improve Vanilla Gradients [27].

In this case, the proposed method consists in creating an image that works as a baseline (generally a completely black image) and interpolating it with the original image to be explained. The gradient is then computed for each step in this interpolation, and all steps are integrated at the end. In this context, interpolation means generating several versions of the image with different levels of transparency, from a totally black image to the original.

According to Sundararajan et al., this process satisfies a series of mathematical axioms regarding sensitivity and invariance, justifying the adoption of this method from a theoretical point of view. They further establish that typically between 20 and 300 interpolation steps are sufficient [27].

## 3. Results and Discussion

By using the procedure described in Section 2.2.4, it was possible to select the best ensembles, that together outperform all other combinations and individual models.

**Figure 3:**
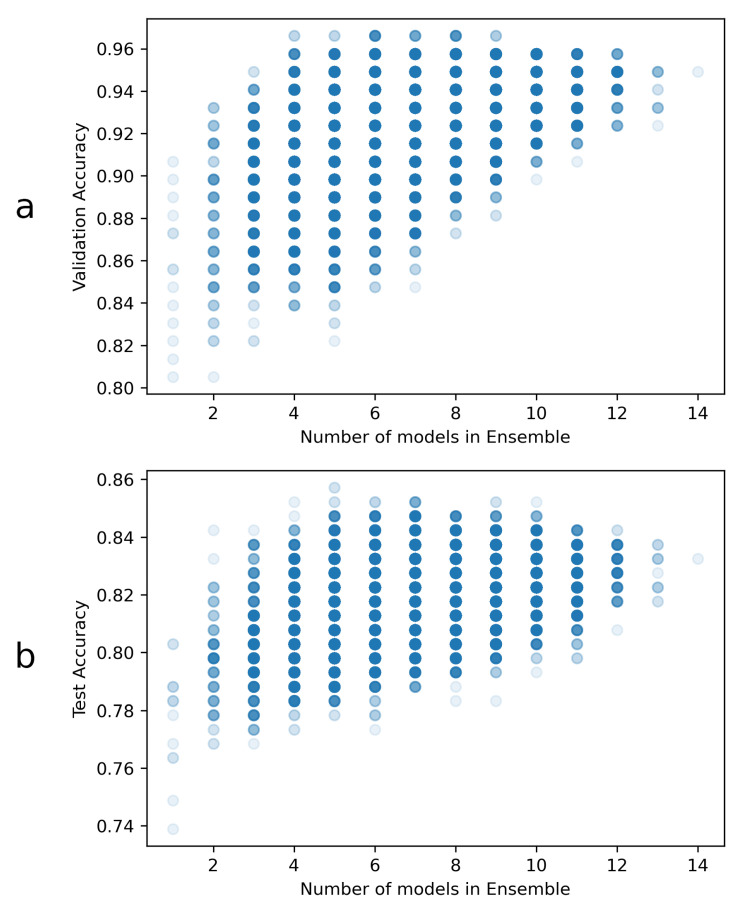
Ensemble accuracies: (**a**) Validation and (**b**) test accuracies, as well as the number of models per ensemble. Each point in these plots might have overlapping ensembles, so the markers have transparency to aid in visualization. A total of 16,383 different ensemble combinations were tested in this way. Notably, the validation results are used for ensemble selection, and the test accuracies are just included here for visualization.

The overall ensemble validation accuracy results are represented in Figure 3. The highest accuracy reached overall is approximately 96.6%, and at least 4 ensembled models were necessary to reach this accuracy. There were a total of three ensembles, composed by 4 models, that achieved this validation accuracy. Since all of them were composed by 2 DenseNets, one VGG and one EfficientNet, one of these 4-model ensembles was chosen at random for the heatmap generation task. The chosen ensemble was formed by the following models:

- VGG16 with validation *acc* of 83.1% (hereinafter referred as $VGG_{val831}$)
- EfficientNet with validation *acc* of 85.6% (hereinafter referred as $Eff_{val856}$)
- DenseNet with validation *acc* of 89.8% (hereinafter referred as $Dense_{val898}$)
- DenseNet with validation *acc* of 90.7% (hereinafter referred as $Dense_{val907}$)

The detailed performance metrics of the 4-models used in this ensemble are shown in Table 1.

Comparing the F1 scores and Accuracy scores achieved for the the training, validation and test sets of each model, it is noted that they were respectively identical up to the third decimal place (any differences observed were smaller than this). That is to say, the train accuracy and train F1 score are identical, as are the validation accuracy and F1 score, and so on.

This result indicates that, although the number of samples in each class is not identical, the differences are small and do not seem to skew the classification. If the dataset was class imbalanced, a possibly observed effect would be higher accuracy values with lower F1 scores, which didn’t happen in this case. However, simply looking at these metrics might give the erroneous impression that the classifier is not biased in any way.

**Figure 4:**
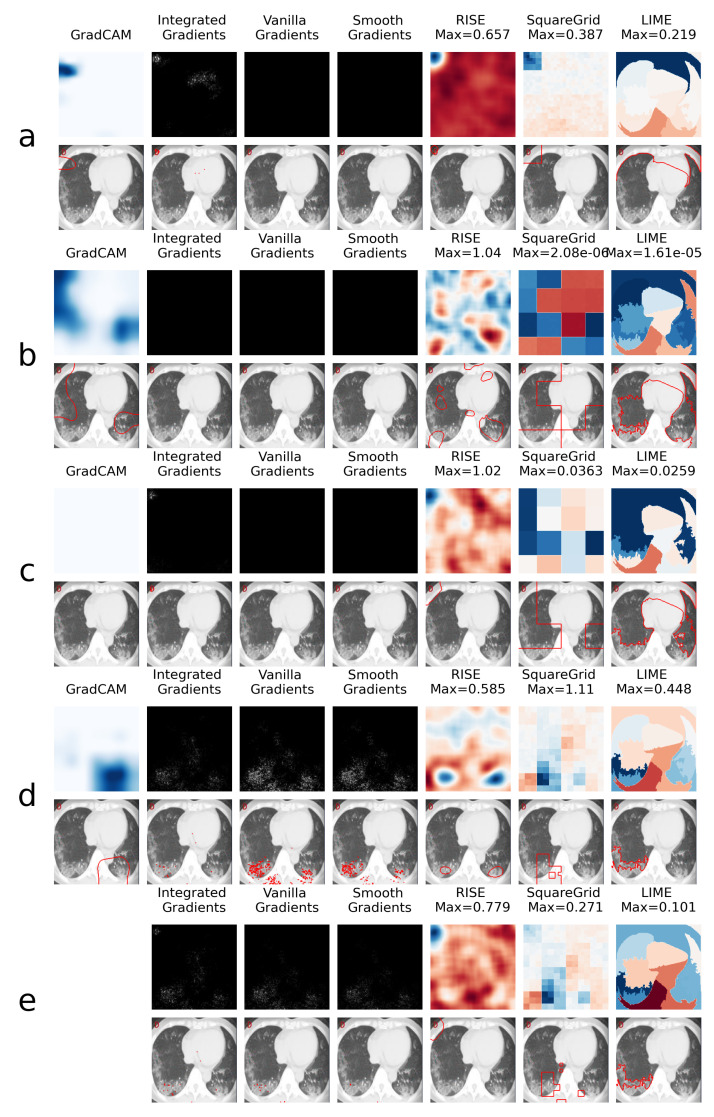
Comparison of XAI heatmaps for image with colored artifact: (**a**) $VGG_{val831}$. (**b**) $Eff_{val856}$. (**c**) $Dense_{val898}$. (**d**) $Dense_{val907}$. (**e**) Ensemble. Top row for each panel shows the heatmaps, while bottom row highlights the areas of highest heatmap weights. Note that GradCAM is not defined for ensembles of models. The scales for the heatmaps go from 0 to 1 for GradCAM, minimum to maximum for RISE and -maximum to maximum for Squaregrid and Gradcam.

**Figure 5:**
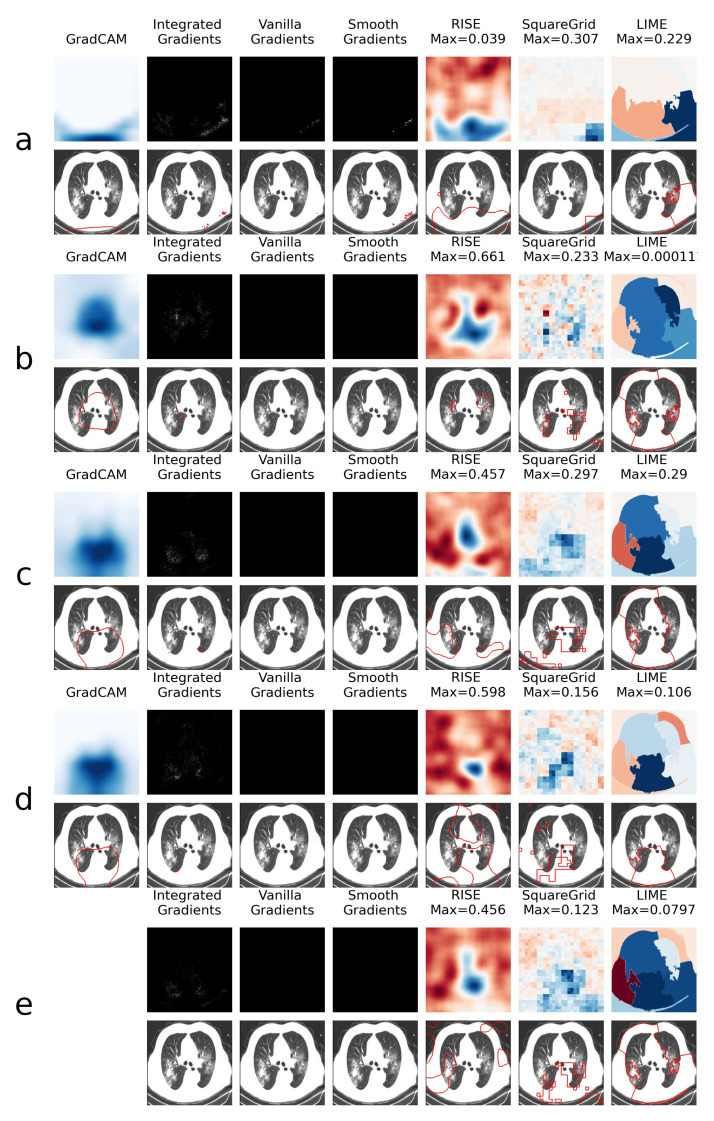
Comparison of XAI heatmaps for image without colored artifacts: (**a**) $VGG_{val831}$. (**b**) $Eff_{val856}$. (**c**) $Dense_{val898}$. (**d**) $Dense_{val907}$. (**e**) Ensemble. See the caption of Figure 4 for details on heatmap scales.

This is explored further through the XAI heatmap generation, as shown in the examples presented in Figure 4 and Figure 5, where different XAI techniques were applied to COVID class images with and without colored artifacts, respectively. For each analyzed image, explanation heatmaps were generated to the classifiers: (a) VGGval831, (b) Effval856, (c) Denseval898, (d) Denseval907 and (e) Ensemble. The heatmaps for LIME and Squaregrid are plotted in a blue/red symmetrical scale going from max to -max, where max is the maximum value of the heatmap. For RISE, the explanation weight signals do not have defined meaning, and the scale goes simply from the maximum (blue) to the minimum (red). The gradient based heatmaps are plotted in a blue colormap for GradCAM (going from 0 to 1, since the map is normalized) and on a greyscale heatmap for the gradients.

For the colored artifact cases (Figure 4), a commonly observed behavior is that $VGG_{val831}$ focuses on the colored artifact (panel **a**). More specifically, considering the particular image depicted in Figure 4, notice that $VGG_{val831}$ focuses on a red letter ‘D’ located on the top left of the image. The GradCAM, Integrated Gradients, RISE, Squaregrid and LIME heatmaps seem to all highlight the region of the image containing the artifact. This is very consistent with the behavior presented by $VGG_{val831}$ for different images throughout the dataset, which seems to be the model most affected by this bias. In turn, panel **a** of Figure 5 (without colored artifacts) shows another typical bias displayed by the VGG model, where it focuses on the bottom of the image, seemingly paying attention to a support structure where the person is laying during the CT-scan. Many images in the COVID class come from papers where this structure is visible, which seems to have created this bias. On the other hand, $Eff_{val856}$ and $Dense_{val898}$ are less affected by these kinds of bias, apparently focusing on the distracting artifacts at times, but on the lung regions in others (Figure 4 and Figure 5, panels **b** and **c**).

Several additional examples of these behaviors, most notably of VGG focusing on seemingly spurious parts of the image regardless of the input, can be seen in the supplemental files provided alongside this manuscript. They include all the images of the dataset which contain letters, as well as images whose classification softmax probabilities surpass 90%.

However, a remarkable behavior can be observed in Figure 4 panels **c** and **d**, both showing DenseNet169 architectures, with a difference of less than 1% in validation accuracy. Although having the same architectures and presenting almost the same validation accuracies, they display very different heatmaps for the image with the colored artifact. $Dense_{val898}$ clearly focuses on the red ’D’, whereas $Dense_{val907}$ focuses on the lungs. Moreover, the GradCAM, SquareGrid and LIME maps go from having very low explanation weights (note the low maximum values and almost completely white GradCAM map), to much higher values in the lung regions.

This seems to show a key non-trivial result: very small increases in validation accuracy can correspond to drastic changes in the concepts learned by the network. In this case, it can mean overcoming a bias introduced by the artifacts. More than 20 additional examples of this behavior can be observed in the supplemental files provided alongside the paper.

Another interesting observation can be made in panel **e**, where the ensemble has a RISE heatmap more similar to $Dense_{val898}$ (focusing on the artifact) and a Squaregrid map more similar to $Dense_{val907}$. This means it’s also not trivial, interpretability-wise, to predict how much each model within an ensemble might contribute to a given explanation. The usage of multiple XAI techniques in tandem allows for probing different aspects of the classifier. In this case, for instance, it would seem that $Dense_{val907}$ is the least biased of the classifiers for the image with the red artifact.

The achieved results indicate that networks could attain high performance metrics on this dataset by focusing on spurious artifacts. Besides, the use of multiple XAI approaches enriches the analysis by showing that models may behave non-trivially when ensembled together.

It also shows that heatmaps generated with different approaches may highlight different regions. This is expected, given that these different premises are akin to asking different questions to the classifier. The more different techniques are used to probe the model, the richer the understanding of the potential biases becomes.

It also seems notable that the VGG16 model used was the most affected by the biases. Although this can’t be generalized beyond this dataset, it suggests further studies should be performed to explore if DenseNet models are more insensitive to biases in general.

## 4. Conclusions

In this study, a number of COVID19 CT-scan classifiers were analyzed using various XAI methods. The models were trained on a dataset that is balanced, considering the number of samples for each classes, but potentially biased by artifacts and spurious aberrations that happen within each class. These clear biases are present in spite of more typical biases, such as those arising from imbalanced classes, not being present (Accuracy, F1 score and AUC metrics of the classifiers are very high). Notably, the VGG16 architecture seemed to be the most affected model in this case.

The results show that XAI methods can be valuable assets for identifying biases in models and comparing how different architectures behave under these types of training conditions. Additionally, the results show that slight improvements in validation accuracy can be associated with drastic and important changes in the concepts learned by the network, to the point of learning or not the biases in the data.

Classifiers such as the ones discussed here typically only provide the softmax probabilities as outputs. Simply looking at those probabilities cannot reliably inform an user whether a CNN is potentially focusing on a spurious part of an image or not. The use of XAI based heatmap approaches is fundamental in this sense.

The results observed also show that different XAI techniques may show the CNN focusing on different parts of the image. This is plausible because the different approaches evaluate different aspects of the classification. Analyzing the gradients back to the last convolution may provide different insights compared to analyzing the gradient back to the input. Similarly, both of these may provide different insights compared to the myriad of ways by which the input can be perturbed.

Overall, these reinforce the importance of employing multiple XAI approaches in tandem, and considering them valuable front-line tools for assessing biases in image classifiers for medical data. This also encourages continuous efforts in creating larger COVID CT-Scan datasets and continuously improving the currently existing ones.

## Figures and Tables

**Table 1:** Performance metrics of the selected ensemble as well as of each of its 4 individual models.

	Accuracy			F1Score			AUC		
**Model**	**Train**	**Val**	**Test**	**Train**	**Val**	**Test**	**Train**	**Val**	**Test**
$VGG_{val831}$	0.871	0.831	0.764	0.871	0.831	0.764	0.948	0.906	0.867
$Eff_{val856}$	0.995	0.856	0.788	0.995	0.856	0.788	1.000	0.884	0.828
$Dense_{val898}$	0.988	0.898	0.803	0.988	0.898	0.803	1.000	0.963	0.880
$Dense_{val907}$	1.000	0.907	0.803	1.000	0.907	0.803	1.000	0.917	0.830
Ensemble	1.000	0.966	0.833	1.000	0.966	0.833	1.000	0.963	0.894

## Data Availability

The four classifier models will be provided on a github repository.

## Supplementary Materials

The following are available online at https://www.dropbox.com/sh/925h7f86o2ooyd8/AAA6iOC1FT32OYUeXijDZ7dta?dl=0.
